# Plasmonic Ag Nanoparticles: Correlating Nanofabrication and Aggregation for SERS Detection of Thiabendazole Pesticide

**DOI:** 10.1021/acsomega.4c07586

**Published:** 2024-10-02

**Authors:** Marcelo J. S Oliveira, Isabela Bianchi-Carvalho, Rafael J. G Rubira, Santiago Sánchez-Cortés, Carlos J. L Constantino

**Affiliations:** † School of Technology and Sciences (FCT), Physics Department, Universidade Estadual Paulista “Júlio de Mesquita Filho” (UNESP), Presidente Prudente 19060-900, São Paulo, Brazil; ‡ Universidade Estadual Paulista “Júlio de Mesquita Filho” (UNESP), Institute of Geosciences and Exact Sciences (IGCE), Physics Department, Rio Claro 13506-900, São Paulo, Brazil; § Instituto de Estructura de la Materia (IEM), Consejo Superior de Investigaciones Científicas (CSIC), 28006 Madrid, Spain

## Abstract

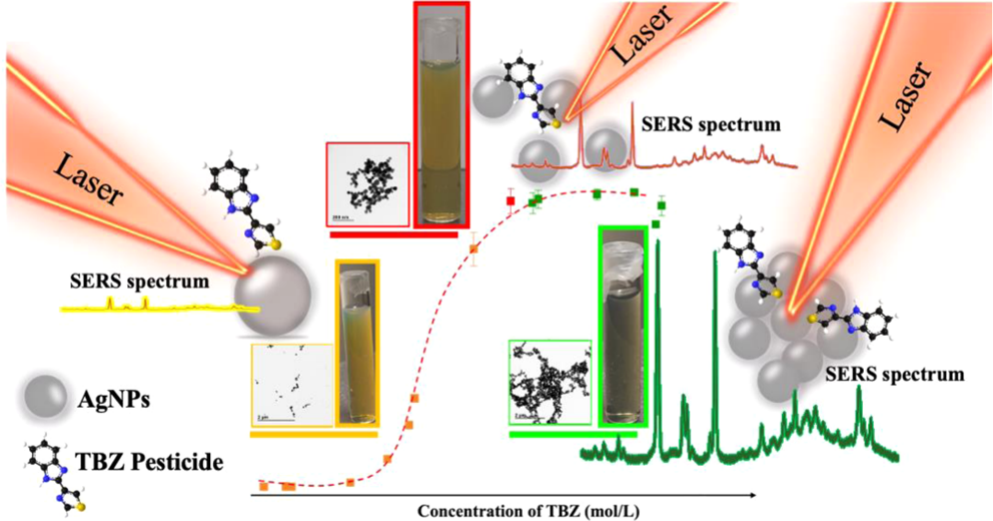

The level of aggregation and aggregate morphology of metallic nanoparticles are factors that influence the SERS signal (surface-enhanced Raman scattering), affecting reproducibility and sensitivity. This study presents a systematic evaluation of the colloidal aggregation on the SERS signal by combining transmission electron microscopy and UV–vis extinction spectroscopy. It focuses on the effect of two methods of sample preparation (“external standard method-ESM” and “standard addition method-SAM”) on the SERS signal using the fungicide thiabendazole (TBZ) in Ag colloid as a probe molecule. The TBZ critical concentration (concentration for which SERS reaches the maximum intensity) was 6.0 × 10^–6^ mol/L for ESM and 1.5 × 10^–6^ mol/L for SAM. Besides, TBZ exhibited a sigmoid-type isotherm for ESM, indicating formation of a TBZ first layer on Ag nanoparticles at lower concentrations (Ag aggregates more compact; size <500 nm) and TBZ multilayers at higher concentrations (Ag aggregates more branched; >2 μm). For SAM, the TBZ first layer formation was also observed at lower concentrations (Ag aggregates more branched; <2 μm). However, at higher concentrations, the Ag colloid degradation/precipitation was observed (Ag aggregates more compact; >2 μm). The Ag aggregation mechanisms align with reaction-limited colloidal aggregation at lower concentrations and diffusion-limited colloidal aggregation at higher concentrations. We believe these results contribute to the SERS research field despite all of the work already done over its 50-year history.

## Introduction

The plasmon hybridization between plasmonic nanoparticles (NPs) is an important electromagnetic plasmon-driven effect that occurs in plasmonic devices and largely determines the resulting Raman intensity in SERS experiments. This hybridization takes place when nanoparticle aggregation is induced by the adsorption of molecules and ions on the interface.^1^ The aggregation is a process where a balance between electrostatic repulsive forces and van der Waals interactions between nanoparticles occurs in NPs. Depending on factors like the nature of the adsorbate, the surface covering, the temperature, and the physicochemical properties of the metal interfaces, the aggregation mechanism of plasmonic nanoparticles can vary from reaction-limited colloid aggregation (RLCA) to diffusion-limited colloid aggregation (DLCA) or vice versa, leading to different morphologies and fractal dimensions of the resulting aggregates.

These mechanisms are discussed here following the approaches of Lin et al.^2−4^ and Sanchez-Cortés et al.^5,6^ and the references therein. Both groups of authors start from common premises, considering the adsorption of the adsorbate (or solute) on colloidal nanoparticles. The adsorbate molecules, neutral in terms of charge, replace the ions that cover the nanoparticles (ions from the synthesis of the colloid), thus reducing the electrostatic repulsion between the nanoparticles and facilitating the aggregation of the colloid. They also agree that, therefore, lower concentrations of adsorbate imply less coating of the surface of the nanoparticles (nanoparticles with higher charge density) and lower colloidal aggregation, whereas the opposite occurs for higher concentrations of adsorbate (nanoparticles with lower charge density). Finally, both groups of authors agree that nanoparticles with lower charge density (higher adsorbate concentrations) lead to faster aggregation, being limited by the diffusion of the growing clusters (DLCA mechanism), while nanoparticles with higher charge density (lower adsorbate concentrations) lead to slower aggregation, being limited by nanoparticle–nanoparticle adhesion (reaction) (RLCA mechanism).

However, Lin et al.^2−4^ have observed that the DLCA mechanism leads to more opened aggregates, while the RLCA mechanism results in more compact aggregates. For instance, they observed that the trend for gold, silica, and polystyrene colloids and using 10^–2^ mol/L of pyridine (adsorbate) was to induce the colloidal aggregation via DLCA mechanism and 10^–4^ mol/L of pyridine to induce the colloidal aggregation via RLCA mechanism. Lin et al.^1^ developed the following approach to define the DLCA and RLCA mechanisms (shortly): the rate of aggregation is determined by the probability, *P*, that two particles will remain together when colliding, where *P* is given by *P* α e^–(Eb/*kT*)^. The height of the resulting repulsive barrier, Eb, must be significantly greater than *kT* for the colloid to remain stable against aggregation. When Eb is reduced, colliding particles can overcome the barrier and stay together, initiating the aggregation process. Considering that the repulsive barrier is completely removed, so that Eb ≪ *kT* and *P* ≈ 1, then each collision results in particles adhering to each other, the rate of aggregation being limited by the time between diffusion-induced collisions (DLCA mechanism). In this case, two particles adhere immediately after contact, whose diffusive motion ensures that the particles always stick to each other at the edges, making the resulting aggregates more open (which we call here “branched aggregates”). Considering that the repulsive barrier is only slightly reduced, such that Eb > *kT* and *P* is very small, then many collisions are required before two particles adhere to each other, the rate of aggregation being limited by the possibilities of collisions (RLCA mechanism). In this case, the probability of adhesion is so low that, on average, two particles can adopt any physically possible binding configuration, making the resulting aggregates more compact (we also called here “compact aggregates”).

On the other hand, Sanchez-Cortés et al.,^5,6^ working with 1,5-dimethylcytosine (DMC) and Ag colloid, observed that the DLCA mechanism (DMC at 10^–3^ mol/L) results in aggregates with more globular morphology (which we call here “compact aggregates”), causing the shift to shorter wavelengths (blue shift) of the aggregate plasmonic band in the UV–vis extinction spectrum. In contrast, the RLCA mechanism (DMC at 10^–6^ mol/L) results in aggregates with more linear morphology (which we call here “branched aggregates”), shifting to longer wavelengths (red shift) their plasmonic band in the UV–vis spectrum. Sanchez-Cortés have also observed a maximum SERS intensity for DMC at 10^–4^ mol/L, where a combination of linear and globular aggregates is found (which we call here the “aggregation transition regime”).

We have a previous work on the thiabendazole (TBZ) pesticide as well using Ag colloid^7^ to activate the SERS effect—a phenomenon whose detailed description can be found in the valuable books by Aroca^8^ and by Le Ru and Etchegoin.^9^ In Oliveira et al.^7^ we focused on the detection of TBZ, trying to build an analytical curve to determine the limit of detection, in which we have succeeded, besides determining the TBZ adsorption mechanism on AgNPs. The results regarding SERS intensity vs TBZ concentration also allowed us to establish the TBZ adsorption isotherm on AgNPs, which was a sigmoid type, as we found here. However, in that previous work, the discussion regarding RLCA and DLCA was not our concern, mainly because of the impossibility of running a detailed study via transmission electron microscopy (TEM). In that work,^7^ we have also observed the red shift of the UV–vis extinction band related to the colloid aggregation, however, with increasing the TBZ concentration, i.e., in an opposite way found for Santiago et al. using DMC. Therefore, in (7) not because of the trend of the UV–vis aggregated band with TBZ concentration but due to the wavelength values themselves, we speculated that the TBZ was inducing more linear Ag aggregates at higher concentrations and more compact aggregates at lower concentrations.

In the present study, we were able to establish a systematic investigation of the Ag colloid aggregation process induced by TBZ combining TEM microscopy and UV–vis extinction spectroscopy and correlate these data with both the DLCA and RLCA aggregation mechanisms and the trend of the SERS signal intensity for different TBZ concentrations. Another element of interest was to monitor the influence of the sample preparation method on both Ag colloid aggregation and SERS activity. In order to check this effect, the TBZ was added to the Ag colloid through two different methods: (a) via external standard method (ESM), and (b) via standard addition method (SAM), ESM being the method applied by Lin et al.,^3^ Sanchez-Cortés et al.,^5^ and ourselves in (7). This review, therefore, offers a systematization that is not found in the literature involving TEM microscopy and UV–vis extinction to explain the SERS results for different adsorbate concentrations and different sample preparation methods. Given the influence of the colloid aggregation on the SERS signal, we believe that these results contribute to the SERS research field despite all of the work already done over its 50-year history.

## Materials and Methods

### Reagents

The thiabendazole fungicide (TBZ–molar mass 201.25 g/mol) was acquired from Sigma-Aldrich. Silver spherical nanoparticles reduced by hydroxylamine (AgNPs) were synthesized from Leopold and Lendl.^10^ Basically, by the latter method, 300 μL sodium hydroxide (1.0 mol/L) was added under agitation to 90 mL hydroxylamine hydrochloride solution (1.6 × 10^–3^ mol/L). Subsequently, 10 mL of silver nitrate (1.0 × 10^–2^ mol/L) was added drop by drop, keeping the colloid under agitation for 10 min to finish the reaction. Due to the low solubility of TBZ in water, the pesticide solutions were prepared from a stock solution of TBZ dissolved in ethanol (10^–2^ mol/L) for subsequent dilutions in an aqueous medium, as illustrated in Figure S1.

### ESM and SAM

**Figure 1:**
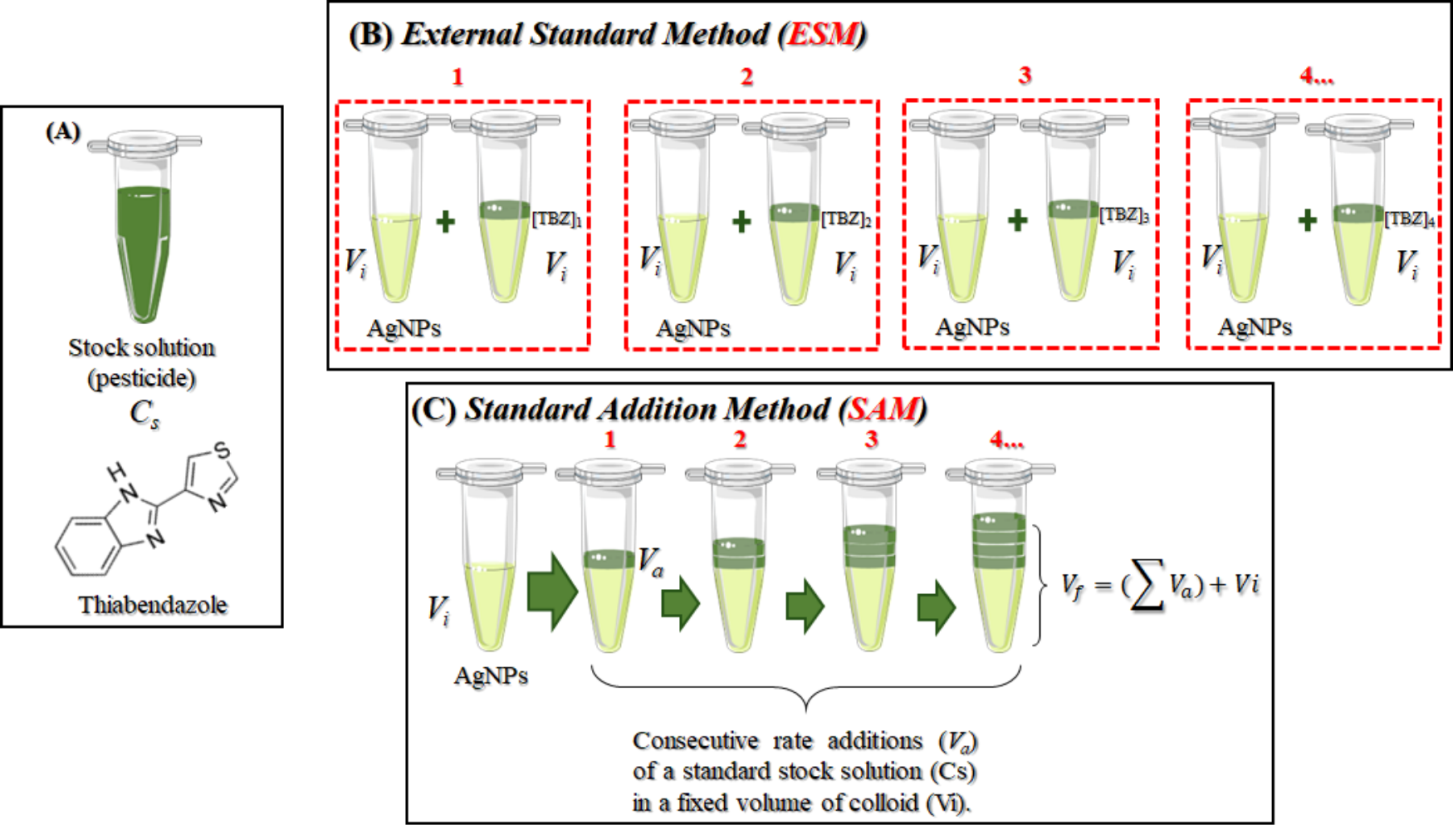
(A) Molecular structure of the pesticide thiabendazole (TBZ) used as the probe molecule (stock solution). Schematic representation of the methods: (B) external standard (ESM) and (C) standard addition (SAM).

The ESM method consists of using a neat colloid (without adding the target molecule previously) for each new addition of TBZ, whereas in the SAM method, several aliquots of TBZ were added each time to the same fixed volume of colloid to give rise to different pesticide concentrations. The measurement is performed after each new addition of TBZ aliquots to the mixture. A representative picture of these methods is displayed in Figure 1.

### Extinction UV–vis, Raman, and SERS Spectra

Extinction UV–vis spectra were recorded for the Ag colloid in the absence and presence of TBZ for both methods (ESM and SAM). The measurements were performed in a spectrophotometer UV–vis–NIR spectrophotometer Shimadzu 3600 equipped with a photomultiplier tube (PMT-photomultiplier tube) for the UV–vis region and InGaAs and PbS detectors for the near-infrared (NIR-near-infrared). The Raman and SERS spectra were recorded using a micro-Raman Renishaw inVia spectrograph equipped with a CCD-cooled detector, a laser line at 785 nm, and diffraction gratings with 1200 l/mm. The Raman spectrum was obtained for TBZ powder, while the SERS spectra were obtained by using the Ag colloid in the presence of different TBZ concentrations for both methods (ESM and SAM). The SERS (ESM) measurements were performed using 10% of the laser power (corresponding to ca. 5 mW) for all concentrations; however, in the SERS (SAM) measurements, only up to 9.7 × 10^–7^ mol/L were made with 10% of the laser intensity. For the other concentrations, 1% of the laser power (0.5 mW) was used due to the saturation of the detector for the band at 240 cm^–1^. Therefore, in the calculations of the analytical curves, the intensities were multiplied accordingly to obtain the same 10% for all measurements. The experiments were performed at least three times, and for each sample of Ag colloid + TBZ, six spectra were obtained. The aliquots employed for each method in both SERS and UV–vis measurements are shown in Table S1 for the ESM measurements and Table S2 for the SAM measurements (Supporting Information). Furthermore, the Ag colloid was previously diluted to avoid detector saturation in the case of the UV–vis measurements.

### TEM

TEM images were obtained at the Universidad Complutense de Madrid using the JEM 1400 Plus equipment, with acceleration voltage from 40 to 120 kV at intervals of 33 V. The TEM mode presents a resolution of 0.38 nm between points and 0.2 nm between lines, with magnification ranging from 50 × to 1.2 × 10^6^×. The camera has a focal length of 15–350 cm in AS DIFF and 4–80 cm in HD DIFF, and the electron cannon is of the thermionic type with a LaB_6_ filament. For the preparation of the samples for the TEM images, the carbon film on 200 mesh copper, acquired from PELCO Pinpointer Grid, was used. Thus, 5 μL of colloidal suspension was deposited on the grid. After 15 min, the remaining excess colloidal suspension was removed, as shown in Figure S2 (Supporting Information). The images were obtained 12 h after sample preparation. To observe the different types of aggregates formed in each method (ESM and SAM), TBZ concentrations were prepared according to the proposed addition method.

## Results and Discussion

### SERS at Different Concentrations: ESM Method

Initially, as shown in Figure S3, the SERS spectra of neat Ag colloid and Ag colloid with 20 μL of ethanol (the maximum amount of ethanol added to the Ag colloid) were obtained, as well as the Raman spectra of ethanol and TBZ in water (10^–4^ mol/L with 1% ethanol, since the stock solution of TBZ at 10^–2^ mol/L is in ethanol).

**Figure 2:**
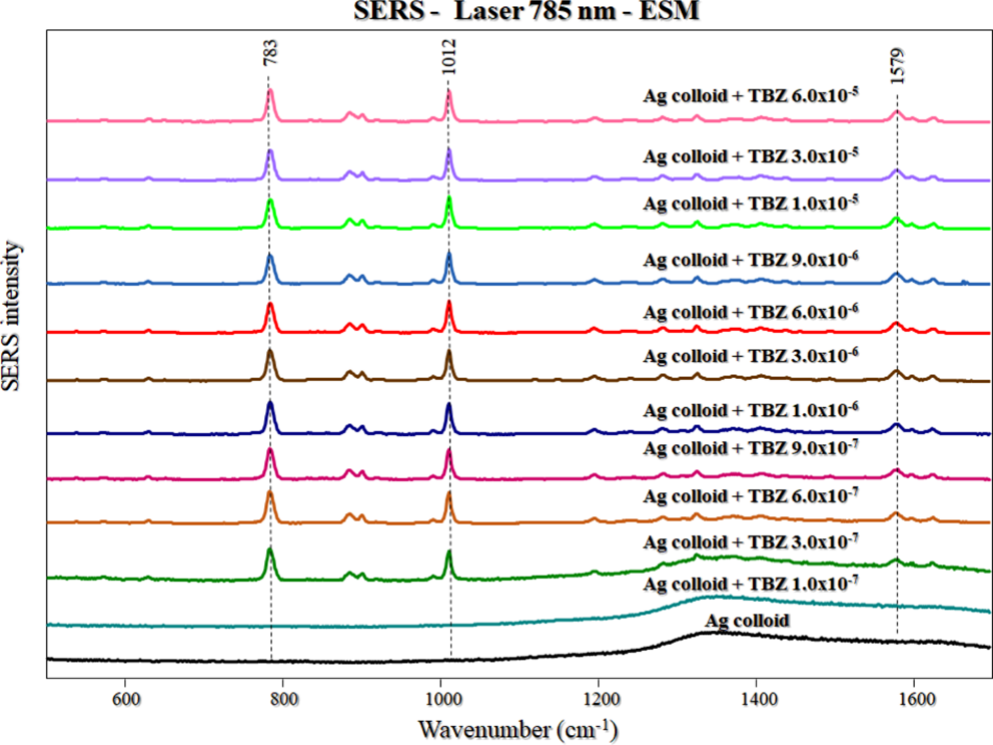
TBZ SERS spectra (Ag colloid) from 1.0 × 10^–7^ to 6.0 × 10^–5^ mol/L. The spectra were plotted with baseline correction and normalization (from 500 to 1700 cm^–1^). Laser line at 785 nm. ESM method.

Figure 2 shows the SERS spectra of TBZ from 1.0 × 10^–7^ to 6.0 × 10^–5^ mol/L for the ESM method. Figure S4 (Supporting Information) shows the SERS spectra of TBZ for all concentrations (from 9.0 × 10^–8^ to 1.0 × 10^–4^ mol/L) tested for the ESM method. It is possible to observe the characteristic peaks of TBZ up to 3.0 × 10^–7^ mol/L (Figure 2), although with low SERS intensity. The most prominent peaks of TBZ are observed at 784 and 1012 cm^–1^ corresponding to the vibrations of S–C and C–H, respectively.^11,12^ According to McCreery,^13^ a Raman signal is considered detectable when the signal-to-noise ratio at the same wavenumber range is greater than or equal to 3. For instance, the calculated ratio value is 15.1 for the peak at 1012 cm^–1^ at 3.0 × 10^–7^ mol/L. However, at 1.0 × 10^–7^ mol/L, the characteristic SERS peaks were no longer observable. According to Desimoni and Brunetti,^14^ the limit of detection (LOD) can be defined as a signal-to-noise ratio of 3, which allows for proving the presence of the analyte in the sample using its characteristic band(s). In our specific case, assuming a linear decrease of the SERS intensity below 3.0 × 10^–7^ mol/L, the estimated lowest detectable signal for TBZ would be 5.9 × 10^–8^ mol/L under the experimental conditions used in this study (785 nm laser line, 10% laser power, and 50×-long lens).

**Figure 3:**
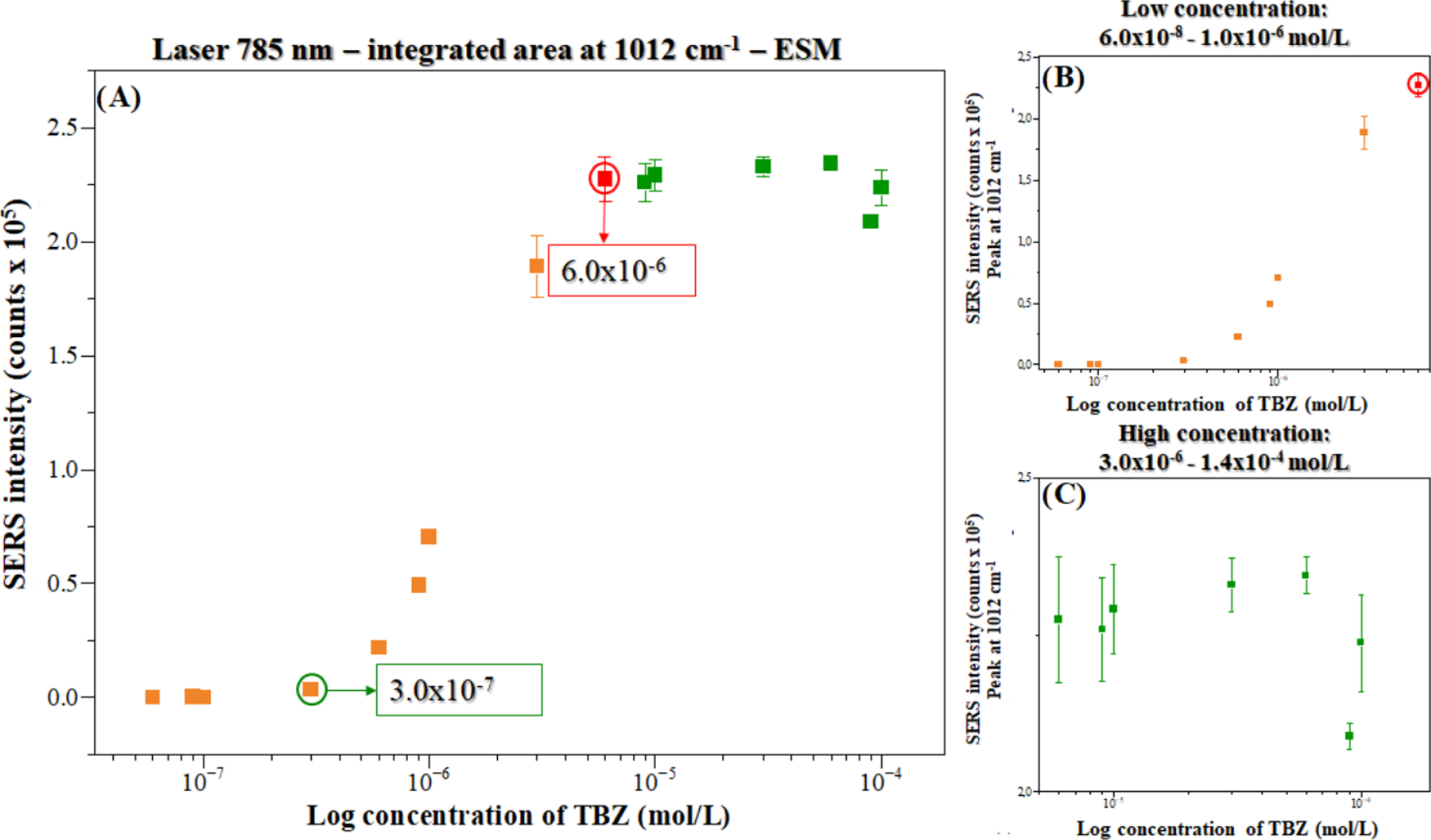
SERS intensity (integrated area for the peak at 1012 cm^–1^) vs log TBZ concentration in (A) all concentrations from 6.0 × 10^–8^ to 1.0 × 10^–4^ mol/L, (B) low concentrations from 6.0 × 10^–8^ to 1.0 × 10^–7^ mol/L, and (C) high concentrations from 3.0 × 10^–6^ to 1.0 × 10^–4^ mol/L. Laser line at 785 nm. ESM method.

To observe the trend of the SERS intensity with TBZ concentration, Figure 3 shows the TBZ adsorption isotherm for the integrated area of the peak at 1012 cm^–1^ vs the TBZ concentration (from 9.0 × 10^–8^ to 1.0 × 10^–4^ mol/L) for all of the spectra shown in Figure S4 (Supporting Information). The results indicate that as the concentration of TBZ increases, the SERS intensity also increases, reaching a plateau at 6.0 × 10^–6^ mol/L (critical concentration). Given that the SERS intensity is proportional to the number of molecules adsorbed on the surface of the nanoparticle, Figure 3A can be considered an adsorption isotherm of TBZ molecules on the surface of the AgNPs.^15^ The sigmoid behavior observed in Figure 3A (ESM method) can be interpreted via the “B.D.D.T model,” named after the initials of the authors of the article who studied different adsorption isotherms, Brunauer, Deming, Deming, and Teller,^16^ which addresses 5 types of adsorption isotherms (Figure S5—Supporting Information) based on the relationship adsorbate–nanoparticle adsorption and adsorbate intermolecular interactions.^17^ These results (sigmoid adsorption isotherm) were repeated twice for the ESM method and are consistent.^7^ Basically, the sigmoid adsorption isotherm indicates the formation of a first layer of TBZ on the surface of the AgNPs at lower concentrations (roughly up to 6.0 × 10^–6^ mol/L—critical concentration), followed by multilayers of TBZ at higher concentrations (plateau).

A similar interpretation of the TBZ adsorption mechanism on the Ag surface, as obtained from the B.D.D.T model for curve V, can be found in the study conducted by Giles et al.,^18^ whose adsorption isotherms are shown in Figure S6, which refers to the mechanism of adsorption of various adsorbates in solution on diverse solid surfaces. Additionally, Giles et al.^18^ provide further insights into the first region of the S-curve (low concentrations), where there is a nonlinear increase in SERS intensity with TBZ concentration (Figure 3B). This suggests that the adsorption of one adsorbate is facilitated by the presence of another adsorbate that was previously adsorbed due to the establishment of intermolecular interactions of different types: ring stacking and H-bonds. The relationships between isotherm types and adsorption mechanisms for TBZ will be further discussed in more detail for the SAM method.

### SERS at Different Concentrations: SAM Method

**Figure 4:**
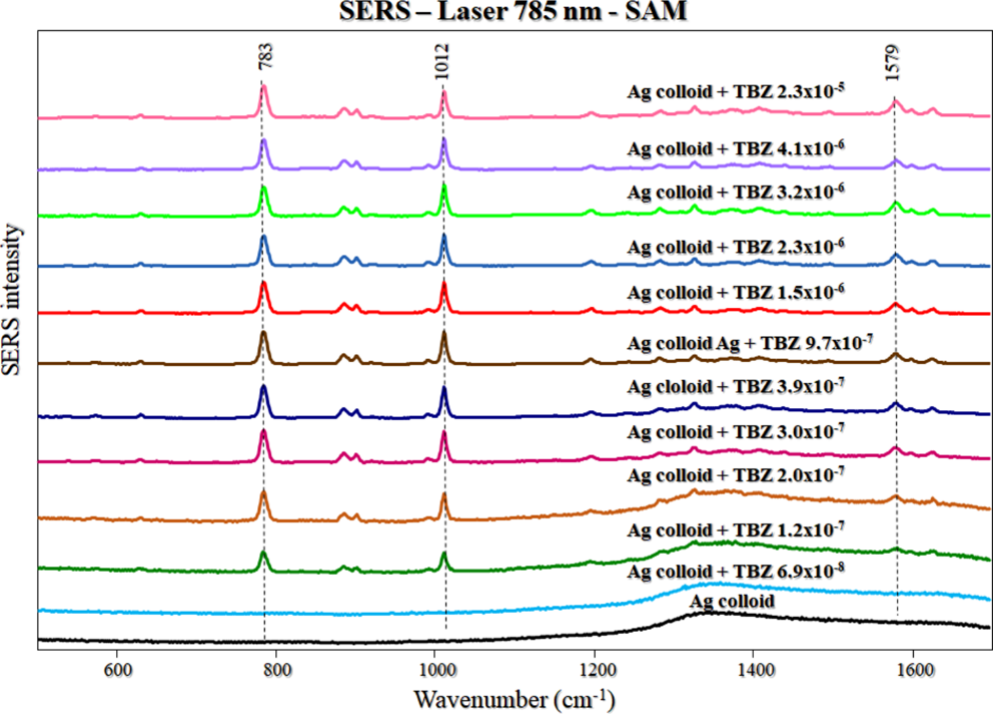
TBZ SERS spectra (Ag colloid) from 6.9 × 10^–8^ to 2.3 × 10^–5^ mol/L. The spectra were plotted with baseline correction and normalization (from 500 to 1700 cm^–1^). Laser line at 785 nm. SAM method.

Figure 4 shows the SERS spectra of TBZ from 6.9 × 10^–8^ to 2.3 × 10^–5^ mol/L for the SAM method. Figure S7 (Supporting Information) shows the SERS spectra of TBZ at all concentrations (from 9.9 × 10^–9^ to 3.2 × 10^–5^ mol/L) tested for the SAM method. In the SERS (SAM) measurements, the characteristic peaks of TBZ were observed at low concentrations from 1.2 × 10^–7^ mol/L. At this concentration, the signal-to-noise ratio of the peak at 1012 cm^–1^ is 7.3. The measurements were conducted using 10% laser power, a 785 nm laser line, and a 50×-long lens. Applying the same assumption used in the ESM method, i.e., assuming that the decrease of the SERS intensity is linear below 1.2 × 10^–7^ mol/L, the LOD for detecting TBZ is estimated to be 4.9 × 10^–8^ mol/L, which is close to the estimated LOD for the ESM method (5.9 × 10^–8^ mol/L here and 6.9 × 10^–8^ mol/L in^7^ using an analytical curve).

**Figure 5:**
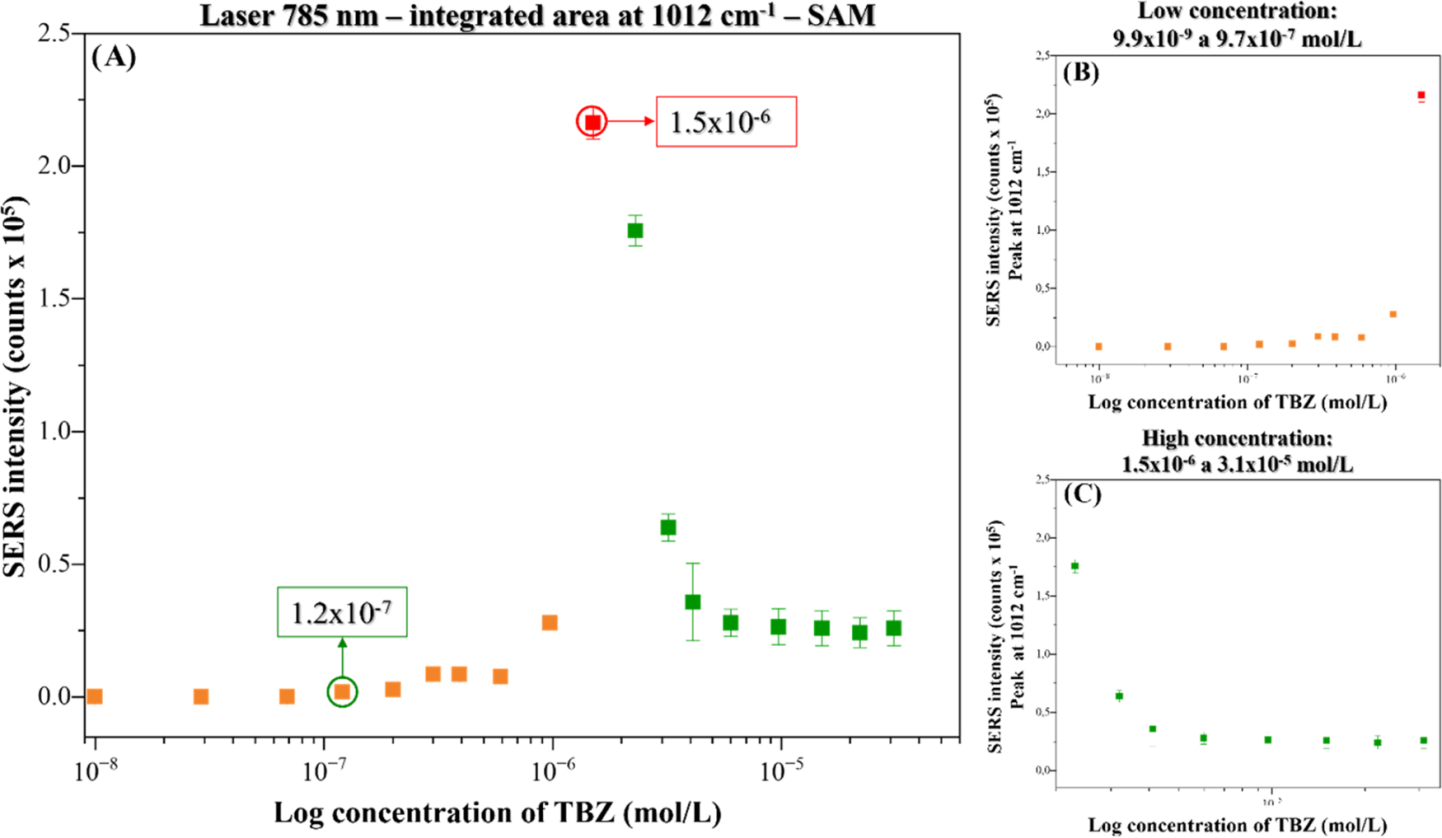
SERS intensity (integrated area of the peak at 1012 cm^–1^) vs log TBZ concentration for (A) all concentrations from 9.9 × 10^–9^ to 3.2 × 10^–5^ mol/L, (B) low concentrations from 9.9 × 10^–9^ to 9.7 × 10^–7^ mol/L, and (C) high concentrations from 1.5 × 10^–6^ to 3.1 × 10^–5^ mol/L. Laser line at 785 nm. SAM method.

Figure 5A shows the TBZ adsorption isotherm from 9.9 × 10^–9^ to 3.2 × 10^–5^ mol/L, with emphasis on regions at low (9.9 × 10^–9^–9.7 × 10^–7^ mol/L-Figure 5B) and high (1.5 × 10^–6^–3.1 × 10^–5^ mol/L-Figure 5C) concentrations. The curve in Figure 5A does not fit any isotherm from the “B.D.DT. model”.^16^ (Figure S5—Supporting Information). However, when the curve is split into low and high concentrations, the curve in Figure 5B (low concentrations) can be associated with curve III in the B.D.D.T. model. Additionally, it can also be associated with the S1 curve reported by Giles et al.,^18^ which describes that in order to obtain curves of the S family (“indicative of the vertical orientation of adsorbed molecules at the surface”), the solute must generally satisfy three conditions: (i) be monofunctional, that is, have a well-defined location of spatial interaction with the substrate; (ii) have moderate intermolecular interaction (allowing the solute molecule to adsorb perpendicular to the substrate surface); and (iii) find strong competition for substrate sites from solvent molecules or another adsorbed species. This implies that in the initial part of the S-curves (low solute concentrations), the more solute that has already been adsorbed, the easier it is to adsorb additional amounts in a “side-by-side” association between adsorbed molecules, helping to keep them on the surface, which has been called “cooperative adsorption”. At this stage, if the orientation of the molecules is such that the new surface has a high attraction to more solute, then the curve rises continuously. In the final stage of the S-curves (high concentrations), as more solute is adsorbed, there is usually less chance that a solute molecule in the solution will find a site for adsorption on the substrate due to a possible saturation of the adsorption on the available TBZ-capped nanoparticles occurring at a concentration of TBZ of 10^–6^ mol/L.

The TBZ is predominantly monofunctional, can anchor to the AgNPs through the S atom preferentially, and has a moderate intermolecular interaction (there is no evidence of strong interaction between the TBZ molecules in solution; in the case of such interaction, for instance, the profile of the UV–vis spectra would vary with the concentration of TBZ, which is not observed in the range considered here from 1.0 × 10^–4^ to 1.0 × 10^–5^). Previous results of our group,^7^ as well as the findings presented here (band at 784 cm^–1^ vibrations S–C), suggest that the TBZ molecule adsorbs perpendicularly to the surface of AgNPs and exhibits strong attraction to the surface of AgNPs. Therefore, as suggested for the ESM method via the B.D.D.T model (isotherm V), and according to Giles et al.^18^ (isotherms S1, or S2 in its initial stage), the TBZ adsorption curve shown in Figure 5B for samples prepared by the SAM method (low concentrations up to 1.5 × 10^–5^ mol/L–critical concentration) is characterized by the formation of a first layer of TBZ from its interaction with the nanoparticle, as found for the ESM method.

However, the curve observed in Figure 5C at higher concentrations than 1.0 × 10^–6^ mol/L does not correspond to any of the systems studied so far that could explain such behavior. In the work of Sánchez-Cortés et al.,^5^ the authors conducted studies using 1,5-dimethylcytosine (DMC) as an aggregating agent in Ag and Cu nanoparticles to analyze the relationship between morphology and SERS results. The authors reported the decrease in intensity of the SERS signal, as shown in Figure 5C, and associated the drop with the type of aggregate formed, which in that case would be globular. In our case, another hypothesis could be associated with the desorption of TBZ molecules on the surface of AgNPs promoted by a TBZ–TBZ interaction, occurring to a higher extent than the TBZ–Ag interactions provided by the SAM method from 1.5 × 10^–6^ mol/L of TBZ (critical concentration). However, we find this hypothesis unreasonable, and it can be completely ruled out based on the extinction spectra in UV–vis discussed later (due to the absence of the TBZ band in such spectra). A plausible hypothesis is an excessive colloid aggregation induced by TBZ via the SAM method, to the point of rendering it unsuitable (leading to degradation/precipitation) for the amplification of the SERS signal. This hypothesis is consistent with the results obtained from extinction spectroscopy in UV–vis and optical images discussed below.

### UV–Vis Extinction Spectroscopy: ESM and SAM Methods

The UV–vis extinction spectra of Ag colloid in the presence of TBZ are presented in the Supporting Information from 1.0 × 10^–8^ to 1.0 × 10^–4^ mol/L using the ESM method (Figure S8A—Supporting Information) and from 6.9 × 10^–8^ to 3.2 × 10^–5^ mol/L for SAM method (Figure S9A—Supporting Information). The UV–vis extinction spectrum of Ag colloid presents the *plasmon* centered at 406 nm referring to isolated nanoparticles or transversal plasma resonances of the aggregates,^19^ being a value close to that reported in the literature for Ag colloid with nanoparticles of spherical morphology.^20^ The spectrum of the TBZ 10^–4^ mol/L solution (light blue in Figures S8A and S9A—Supporting Information) shows only a maximum absorption at 297 nm, attributed to the π–π* electronic transition of the aromatic ring.^21^

Figure S8A (Supporting Information–ESM method) shows a continuous decrease of the *plasmon* intensity at 406 nm up to 6.0 × 10^–6^ mol/L of TBZ (critical concentration), for which a more significant decrease is observed, accompanied by a more evident increase of a band at longer wavelengths (>600 nm) corresponding to the new plasmon resonance due to the interparticle hybridization. These changes in the UV–vis extinction spectra indicate the aggregation of the Ag colloid induced by TBZ.^22^ This trend is accentuated at 9.0 × 10^–6^ mol/L, what we call “aggregation transition regime”, and at higher TBZ concentrations, the intensities of both the *plasmon* at 406 nm and the *plasmon* of the aggregates (>600 nm) exhibit variations within a range as the concentration of TBZ increases, tending to a plateau, as shown in Figures S8B,C (Supporting Information—ESM method), respectively. This may be a consequence of excessive aggregation of the Ag colloid, as suggested by its color change from yellowish at 1.0 × 10^–6^ mol/L to grayish at 3.0 × 10^–6^ mol/L, as shown in Figure S10A (Supporting Information). Finally, from 3.0 × 10^–5^ mol/L, the TBZ band appears at 297 nm, indicating that TBZ is no longer adsorbing on AgNPs and their aggregates, remaining as an excess in the colloidal dispersion. The inset in Figure S8A shows the increase of the absorbance at 297 nm vs TBZ concentration.

In Figure S9A (Supporting Information—SAM method), a similar ESM trend is observed with the decrease of the band at 406 nm and the appearance of the band >600 nm as the concentration of TBZ increases. However, these changes occur at different TBZ concentrations: from 6.9 × 10^–8^ to 9.7 × 10^–7^ mol/L, there is a continuous decrease of the band at 406 nm; at 1.5 × 10^–6^ mol/L (critical concentration), there is a significant reduction of the *plasmon* extinction at 406 nm and the emergence of the *plasmon* of the aggregates >600 nm, followed by the “aggregation transition regime” at 2.3 × 10^–6^ mol/L for the SAM method (9.0 × 10^–6^ mol/L for the ESM method), indicating a strong colloidal aggregation induced by TBZ. Moreover, in the SAM method, unlike the ESM one, above 2.3 × 10^–6^ mol/L, as the concentration of TBZ increases, both the *plasmon* at 406 nm (Figure S9B—Supporting Information) and the plasmon of the aggregates >600 nm continue to decrease (Figure S9C—Supporting Information), i.e., the entire spectrum is shifted downward (Figure S9A—Supporting Information). This suggests degradation of the Ag colloid by aggregation excess, which is consistent with the color changes of this colloid, as shown in the optical images in Figure S10B (Supporting Information). The Ag colloid goes from yellowish at 1.5 × 10^–6^ mol/L (critical concentration) to greenish at 2.3 × 10^–6^ mol/L (aggregation transition regime) and starts losing color for higher TBZ concentration until becoming practically colorless, being possible to observe (by naked eyes) aggregates of AgNPs dispersed in the colloid. However, in the SAM method, Figure S9 (Supporting Information), the TBZ band at 297 nm was not observed relative to its excess in the colloidal dispersion, as observed for the ESM method (the highest TBZ concentration used in the SAM method was 3.2 × 10^–5^ mol/L while such band was initially observed from 3.0 × 10^–5^ mol/L in the ESM method).

### TEM Images: ESM Method

**Figure 6:**
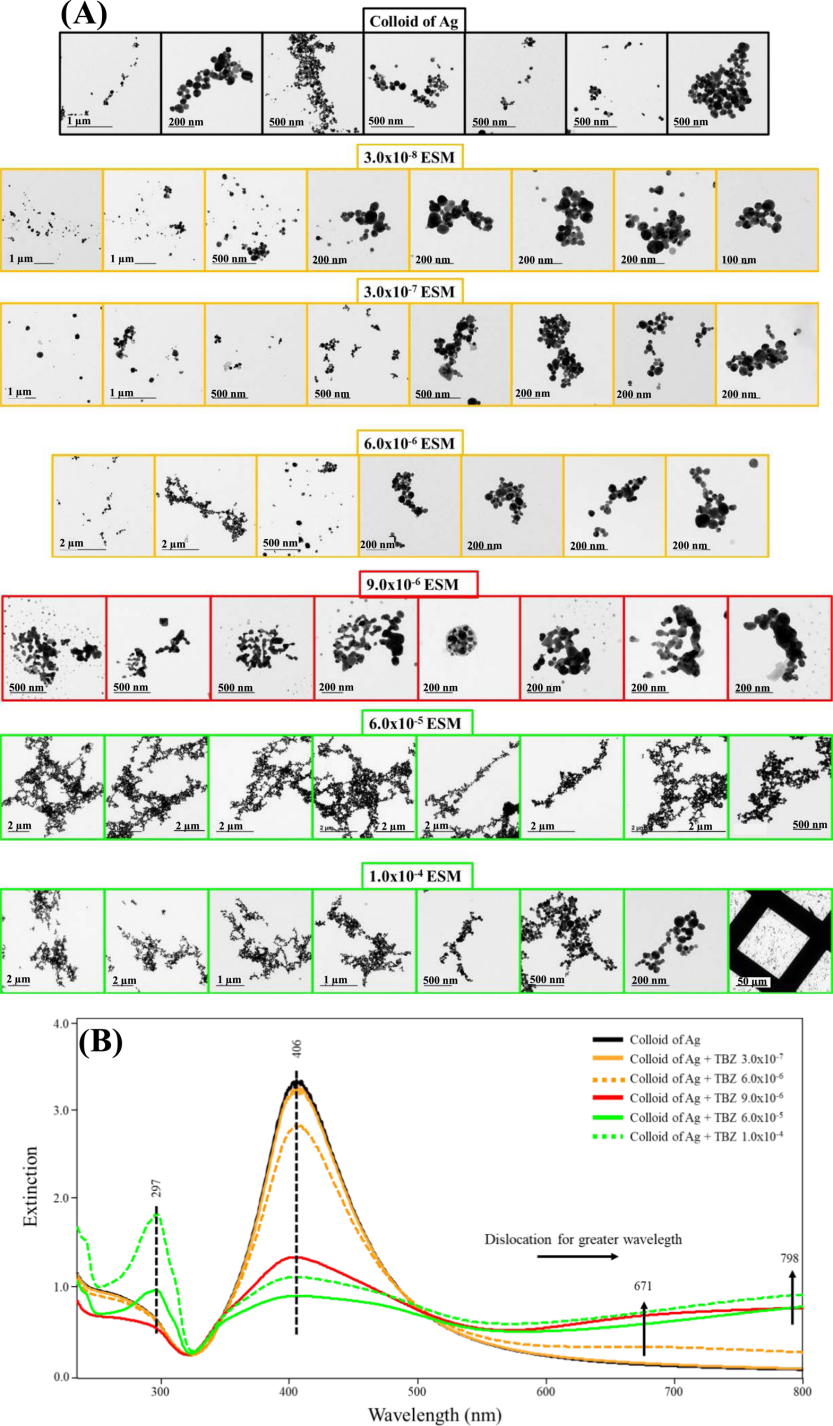
(A) TEM images of the Ag colloid in the absence and presence of TBZ at the same concentrations as the UV–vis extinction spectra. (B) UV–vis extinction spectra of the Ag colloid in the absence (black) and presence of TBZ at low concentrations 3.0 × 10^–8^, 3.0 × 10^–7^, and 6.0 × 10^–6^ mol/L (yellow) at transition concentration 9.0 × 10^–6^ mol/L (red) and at high concentrations 6.0 × 10^–5^ and 1.0 × 10^–4^ mol/L (green). ESM method.

Figure 6A presents TEM microscopy images with their respective UV–vis extinction spectra in Figure 6B for the ESM method. The images were recorded at low concentrations (3.0 × 10^–8^, 3.0 × 10^–7^, and critical concentration at 6.0 × 10^–6^ mol/L), at 9.0 × 10^–6^ mol/L (aggregation transition regime), and at high concentrations (6.0 × 10^–5^ and 1.0 × 10^–4^ mol/L) of TBZ. In Figure 6A, the TEM images of the Ag colloid in the absence of TBZ show the AgNPs with diameters between 40 and 70 nm, in addition to the formation of some aggregates, which may have occurred due to the preparation (drying) of the sample in the *grid* or even generated spontaneously in the dispersion. The UV–vis extinction spectrum of the neat Ag colloid is characteristic of nonaggregated colloid (predominantly).

The TEM images of AgNP aggregates when TBZ concentrations are below the critical concentration at 6.0 × 10^–6^, 3.0 × 10^–8^, 3.0 × 10^–7^, 6.0 × 10^–6^, 1.0 × 10^–7^, and 6.0 × 10^–6^ mol/L (Figures 6 and S11) reveal that the AgNP aggregates formed are, in general, more compacted (globular aggregates), with size <500 nm. Their respective UV–vis extinction spectra show only the gradual decrease of the *plasmon* at 406 nm, except for the critical concentration at 6.0 × 10^–6^ mol/L, which, in addition to this decrease, has the emergence of the *plasmon* band of the AgNP aggregates >600 nm, already suggesting a more significant aggregation of the Ag colloid, as previously mentioned.

In the UV–vis extinction spectrum for Ag colloid containing TBZ at 9.0 × 10^–6^ mol/L (aggregation transition regime), the decrease of the *plasmon* band at 406 nm is abrupt (about 55%), with an increase in extinction intensity at 671 nm (almost 3 times).

At high concentrations of TBZ (6.0 × 10^–5^ and 1.0 × 10^–4^ mol/L) in the Ag colloid, the TEM images in Figure 6A show aggregates with more branched shapes and larger than 2 μm, in general. The UV–vis spectra of the Ag colloid for these TBZ concentrations show a gradual decrease of the *plasmon* band at 406 nm; the increase of the aggregate *plasmon* band >600 nm. In addition, the latter maximum of this high wavelength maximum moves from 631 to 798 nm, thus indicating an increase of the size of the aggregates. Furthermore, the emergence of the free TBZ band in the colloidal dispersion (TBZ not adsorbed to AgNPs and their aggregates) at 297 nm was seen, as previously discussed.

### TEM Images: SAM Method

**Figure 7:**
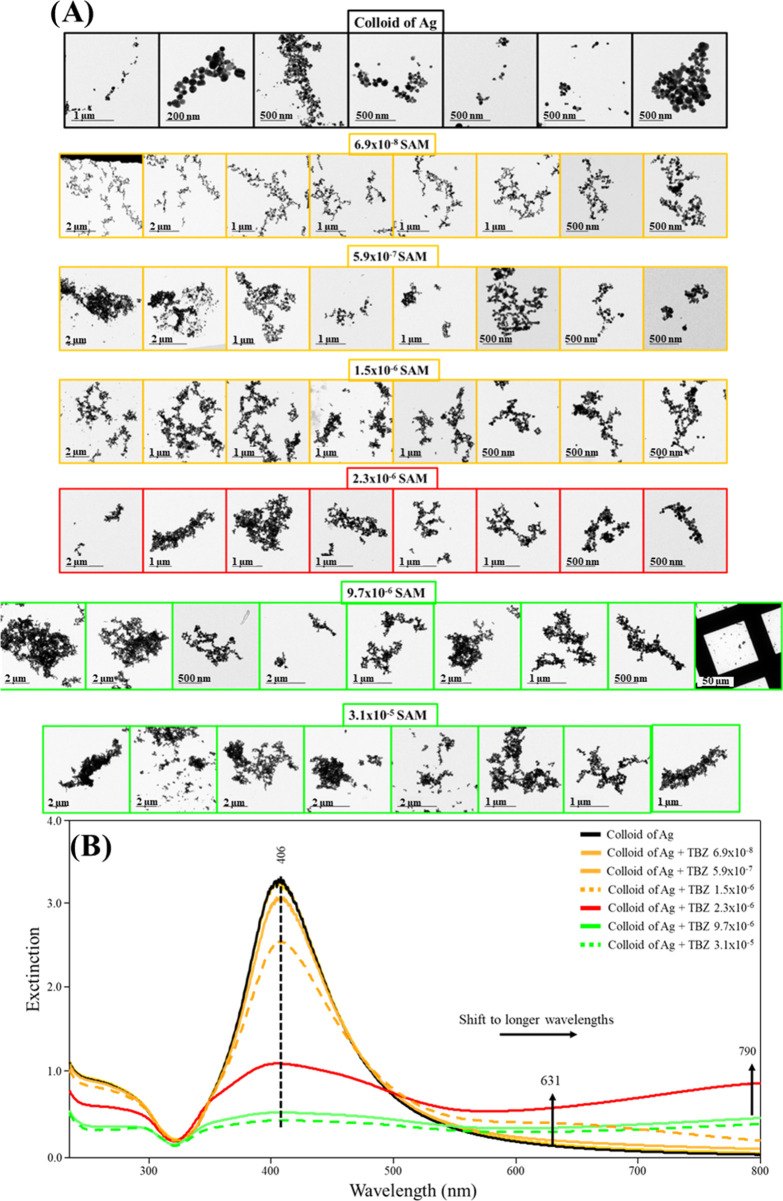
(A) TEM images of the Ag colloid in the absence and presence of TBZ at the same concentrations as in the UV–vis extinction spectra. (B) UV–vis extinction spectra of the Ag colloid in the absence (black) and presence of TBZ at low concentrations 6.9 × 10^–8^, 5.9 × 10^–7^, and 1.5 × 10^–6^ mol/L (yellow) at transition concentration 2.3 × 10^–6^ mol/L (red) and at high concentrations 9.7 × 10^–6^ and 3.1 × 10^–5^ mol/L (green). SAM method.

The TEM images in Figure 7A (and Figure S12) for the SAM method show more branched aggregates at low concentrations (6.9 × 10^–8^, 5.9 × 10^–7^, and critical concentration at 1.5 × 10^–6^ mol/L) in comparison to those observed for the ESM method, and sizes up to 2 μm, in general. In addition, the UV–vis extinction spectra in Figure 7B follow a decrease of the *plasmon* band at 406 nm and the emergence of the aggregate *plasmon* band >600 nm at a much lower adsorbate concentration (2.3 × 10^–6^ mol/L). TEM images of the AgNPs at the latter aggregation transition regime (2.3 × 10^–6^ mol/L) are shown in Figure 7A and reveal the appearance of more compacted aggregates, with the UV–vis extinction spectrum of 2.3 × 10^–6^ mol/L of TBZ presenting an abrupt decrease (about 66%) of the *plasmon* band at 406 nm with the maximum of the aggregate *plasmon* band being shifted from 631 to 790 nm (Figure 7B). This is attributed to the much more extensive aggregation obtained in the SAM method, leading to the appearance of large aggregates in suspension (Figures S10 and S12).

At high concentrations (9.7 × 10^–6^ and 3.1 × 10^–5^ mol/L), the TEM images in Figure 7A show that the aggregates become relatively more compacted, with dimensions greater than 2 μm, unlike the trend observed for the ESM method. The UV–vis extinction spectra in Figure 7B for these TBZ concentrations, however, unlike those observed for the ESM method, drop in intensity for all wavelengths, suggesting the degradation/precipitation of the Ag colloid, as previously discussed.

### General Discussion

Making a general analysis of the UV–vis extinction spectra and TEM images (besides optical microscopy), it is clear that there is a correlation between the changes in the Ag colloid induced by TBZ and the intensity pattern of the TBZ SERS signal as a function of TBZ concentration in the Ag colloid. Remember that in the ESM method, the TBZ molecules find the AgNPs without any preadsorbed TBZ molecule for each TBZ aliquot added, while in the SAM method, except for the first TBZ aliquot, for the others, the TBZ already finds the AgNPs with some TBZ coating, adsorbed on the previous aliquots. In addition to a possible start of aggregation depending on the concentration of TBZ, we found the following for both methods.

In the ESM method, the transition of the morphology of the AgNPs in terms of size and shape of their aggregates, considering TBZ concentrations below and above 9.0 × 10^–6^ mol/L (aggregation transition regime), goes from more compacted AgNP aggregates with dimensions <500 nm to more branched AgNP aggregates with dimension >2 μm, leading to a stabilization of the intensity of the SERS signal, which reaches a plateau. Consequently, the SERS intensity shows a relatively slow increase for the initial contractions of TBZ, then undergoes a rapid increase up to TBZ concentrations between 3.0 × 10^–7^ and 6.0 × 10^–6^ mol/L (critical concentration), reaching a plateau thereafter. This indicates the formation of a sigmoid adsorption isotherm, suggesting the initial layer of TBZ forms on the surface of the AgNPs at low concentrations, while multiple layers of TBZ form at higher concentrations. Besides, it also indicates that more compacted AgNP aggregates with dimensions up to 500 nm seem to be more suitable to activate the SERS effect using TBZ and Ag colloid for the ESM method.

In the SAM method, the transition of the morphology of the AgNPs in terms of size and shape of their aggregates, considering TBZ concentrations below and above 2.3 × 10^–6^ mol/L (aggregation regime transition), goes from more branched AgNP aggregates with dimensions <2 μm to more compacted AgNP aggregates with dimension >2 μm. However, differently from what was found for the ESM method, there was a degradation of the Ag colloid, implying the precipitation of AgNP aggregates and, consequently, the significant decrease of the SERS signal after reaching a maximum intensity at 1.5 × 10^–5^ mol/L (critical concentration). Therefore, considering the SAM method for TBZ and Ag colloid, the more branched aggregates with dimensions up to 2 μm seem to be more suitable to activate the SERS effect.

In terms of morphology, it is noted that in the ESM method, there is an aggregation transition regime at a TBZ concentration of 9.0 × 10^–6^ mol/L. At lower concentrations, the shape of the AgNP aggregates is more compacted due to the aggregation via the RLCA mechanism (metallic nanoparticles with higher surface charge density). In contrast, at concentrations above the critical concentration, the formation of more branched AgNP aggregates is seen. This means that at the latter conditions, the AgNP aggregation proceeds via the DLCA mechanism (metallic nanoparticles with lower surface charge density).

Regarding the SAM method, it was observed that the opposite pattern was found for the ESM method, i.e., at higher TBZ concentrations (above aggregation transition regime at 2.3 × 10^–6^ mol/L), more compacted AgNP aggregates are formed. At lower TBZ concentrations, more branched AgNP aggregates are formed. However, it must be noted that the statement regarding the dependence of the DLCA and RLCA mechanisms on the adsorbate concentration applied here refers to only the ESM method; i.e., only in the ESM method are the premises of Lin et al. met, as the kinetics allow establishing an equilibrium between the adsorbate bound to the metal and the nonadsorbed one. In the SAM method, this kinetics is lacking, and therefore, a deviation from the method is observed.

## Conclusions

SERS intensity strongly depends on the morphology of AgNP aggregates, but the size and shape of these aggregates depend on processes such as the adsorption of the analyte and the aggregation regime. The aggregation process is the critical event that determines the formation of spots upon interparticle hybridization and will assess the final SERS performance of the nanostructured platform employed in SERS detection. One important conclusion of the present work is the fact that the experimental methodology (ESM vs SAM) must be carefully considered in the application of the correct methodology. ESM provides more reliable, stable, and reproducible results in comparison to the SAM. Interestingly, there is a critical concentration, higher in the case of the ESM method (ca. 6 × 10^–6^ mol/L), at which a transition between the RLCA to the DLCA aggregation mechanisms is taking place, while in SAM, this transition is opposite and it occurs at a lower critical concentration (ca. 1.5 × 10^–6^ mol/L). In general, branched aggregates have proven to be more suitable for SERS detection, and it would be interesting to determine the experimental conditions under which these morphologies form for each SERS experiment and for each molecule.

## Supporting Information Available

Table of concentrations of TBZ stock solution; sample preparation for TEM images; SERS spectra (ESM and SAM methods); adsorption isotherms; isothermal classification system; UV–vis extinction spectra (ESM and SAM methods); sample photos; TEM images (ESM and SAM methods) (PDF)
