# Supplementary material for: Plasmonic Ag Nanoparticles: Correlating Nanofabrication and Aggregation for SERS Detection of Thiabendazole Pesticide
Source: ACS Omega. 2024 Oct 2;9(41):42571–81. doi: 10.1021/acsomega.4c07586 (PMC11483382; doi:10.1021/acsomega.4c07586)
Supplement: Supplementary file 1 — ao4c07586_si_001.pdf [file ao4c07586_si_001.pdf]

Plasmonic Ag Nanoparticles: Correlating Nanofabrication and Aggregation for SERS Detection of Thiabendazole Pesticide

Marcelo J. S. Oliveira[*1] ‡, Isabela Bianchi-Carvalho[1] ‡, Rafael J. G. Rubira[2],*

Santiago Sánchez-Cortés[3], Carlos J. L. Constantino[*1]*

[1]Universidade Estadual Paulista "Júlio de Mesquita Filho" (UNESP), School of Technology and Sciences (FCT), Physics Department, 19060-900, Presidente Prudente, SP, Brazil.

[2]Universidade Estadual Paulista "Júlio de Mesquita Filho" (UNESP), Institute of Geosciences and Exact Sciences (IGCE), Physics Department, 13506-900, Rio Claro, SP, Brazil.

[3]Instituto de Estructura de la Materia (IEM), Consejo Superior de Investigaciones Científicas (CSIC), 28006, Madrid, Spain.

Aggregation mechanism, thiabendazole, SERS, detection

Table S1: concentrations of the TBZ stock solutions and the respective final concentrations of "Ag colloid + TBZ" used in the SERS measurements. **ESM method**.

Stock solution of TBZ (mol/L)	Total volume of TBZ added to the Ag colloid	Total sample volume (Ag colloid + TBZ) (µL)	Final concentration of TBZ in Ag colloid (mol/L)
$5.0x10^{-7}$	20	1000	$1.0x10^{-8}$
$1.5x10^{-6}$	20	1000	$3.0x10^{-8}$
$3.0x10^{-6}$	20	1000	$6.0x10^{-8}$
$4.5x10^{-6}$	20	1000	$9.0x10^{-8}$
$5.0x10^{-6}$	20	1000	$1.0x10^{-7}$
$1.5x10^{-5}$	20	1000	$3.0x10^{-7}$
$3.0x10^{-5}$	20	1000	$6.0x10^{-7}$
$4.5x10^{-5}$	20	1000	$9.0x10^{-7}$
$5.0x10^{-5}$	20	1000	$1.0x10^{-6}$
$1.5x10^{-4}$	20	1000	$3.0x10^{-6}$
$3.0x10^{-4}$	20	1000	$6.0x10^{-6}$
$4.5x10^{-4}$	20	1000	$9.0x10^{-6}$
$5.0x10^{-4}$	20	1000	$1.0x10^{-5}$
$1.5x10^{-3}$	20	1000	$3.0x10^{-5}$
$3.0x10^{-3}$	20	1000	$6.0x10^{-5}$
$4.5x10^{-3}$	20	1000	$9.0x10^{-5}$
$5.0x10^{-3}$	20	1000	$1.0x10^{-4}$

Table S2: concentrations of the TBZ stock solutions and the respective final concentrations of "Ag colloid + TBZ" used in the SERS measurements. **SAM method**.

Stock solution of TBZ (mol/L)	Added volume of TBZ (µL) to the Ag colloid	Total volume of TBZ added to the Ag colloid	Total sample volume (Ag colloid + TBZ) (µL)	Final concentration of TBZ in Ag colloid (mol/L)
**1.0×10^{-5}**	1	1	1001	9.9×10^{-9}
	2	3	1003	2.9×10^{-8}
	4	7	1007	6.9×10^{-8}
	6	13	1013	1.2×10^{-7}
	8	21	1021	2.0×10^{-7}
	10	31	1031	3.0×10^{-7}
**1.0×10^{-4}**	1	1	1032	3.9×10^{-7}
	2	3	1034	5.9×10^{-7}
	4	7	1038	9.7×10^{-7}
	6	13	1044	1.5×10^{-6}
	8	21	1052	2.3×10^{-6}
	10	31	1062	3.2×10^{-6}
**1.0×10^{-3}**	1	1	1063	4.1×10^{-6}
	2	3	1065	6.0×10^{-6}
	4	7	1069	9.7×10^{-6}
	6	13	1075	1.5×10^{-5}
	8	21	1083	2.2×10^{-5}
	10	31	1093	3.1×10^{-5}

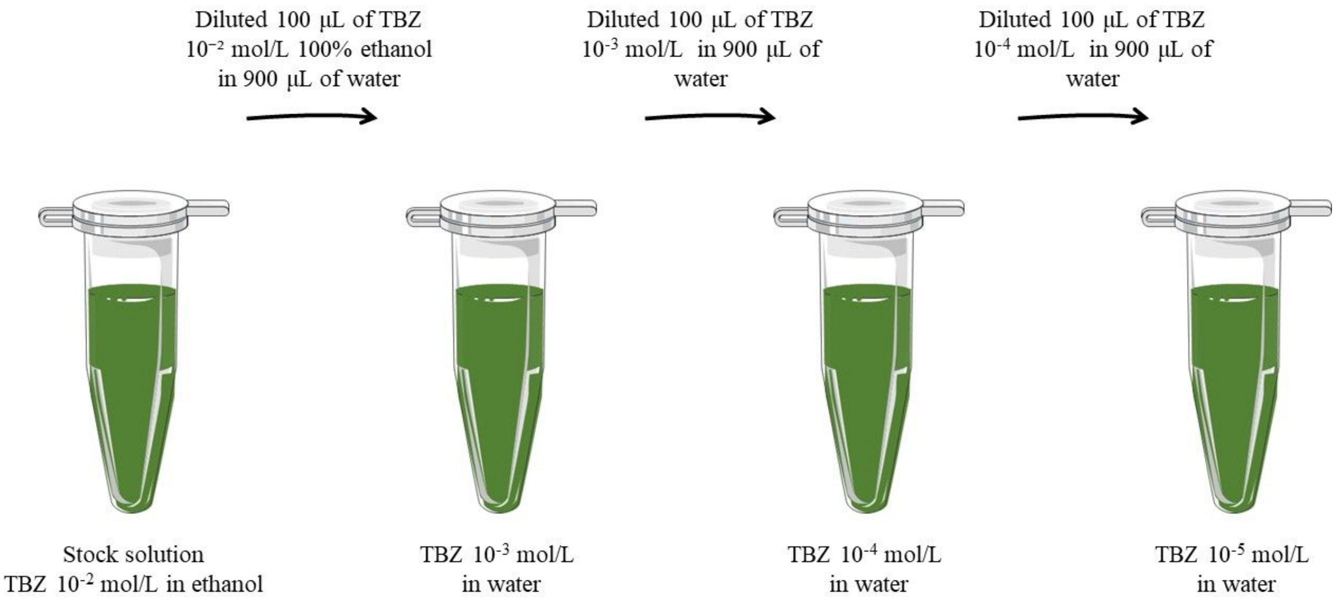

Figure S1: preparation of TBZ dilutions at various concentrations (10^{-2}, 10^{-3}, 10^{-4}, and 10^{-5} mol/L). For 10^{-2} mol/L solution, TBZ is dissolved in ethanol. The 10^{-3} mol/L solution contains 10% ethanol in water, 10^{-4} mol/L contains 1% ethanol, and 10^{-5} mol/L contains 0.1% ethanol. These TBZ solutions will still be diluted in Ag colloid.

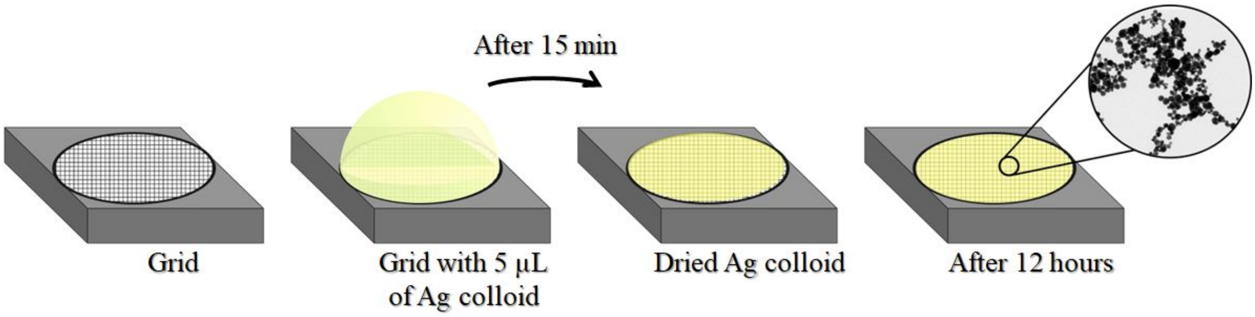

Figure S2: illustration of the sample preparation to obtain the TEM images.

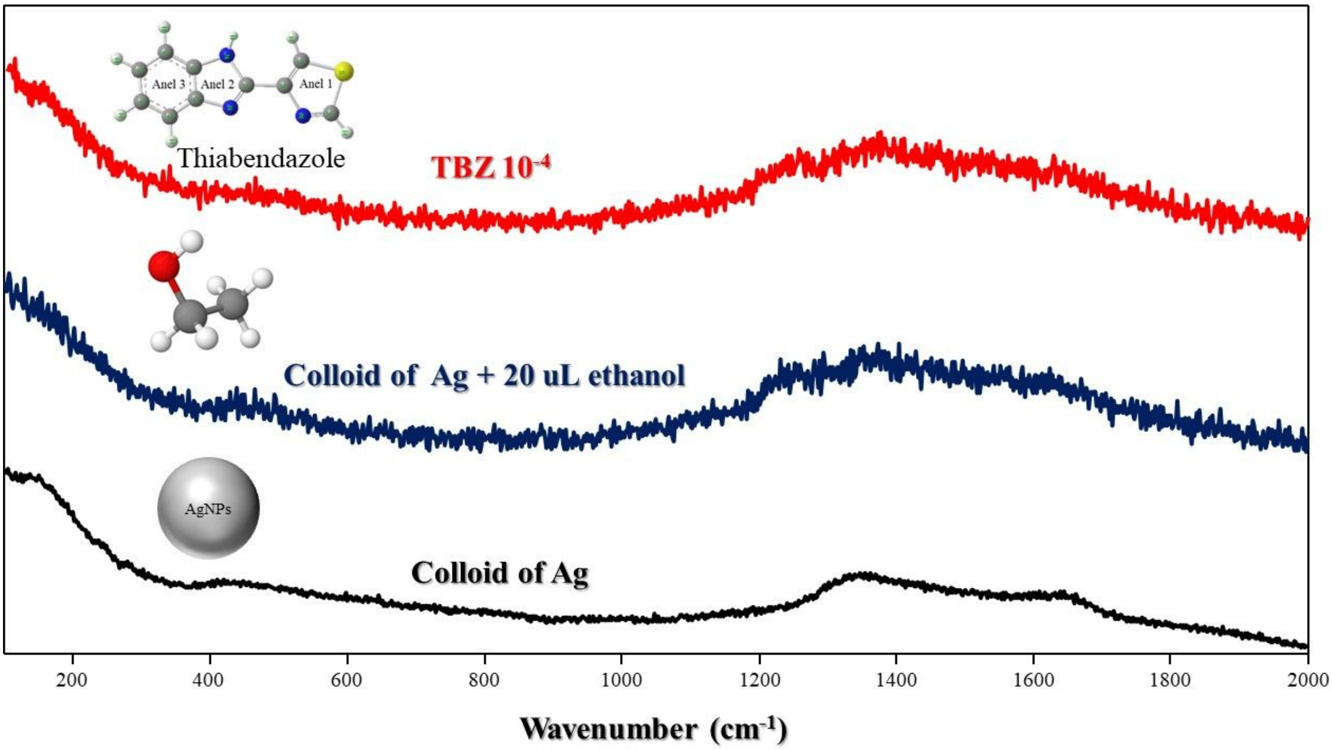

Figure S3: SERS spectra of neat Ag colloid in the presence and absence of 20 µL of ethanol and Raman spectra of TBZ in water and 1% ethanol (10^{-4} mol/L). Laser: 785 nm. Spectra recorded under the same experimental conditions, with baseline correction, and normalized (the most intense band with the same "height").

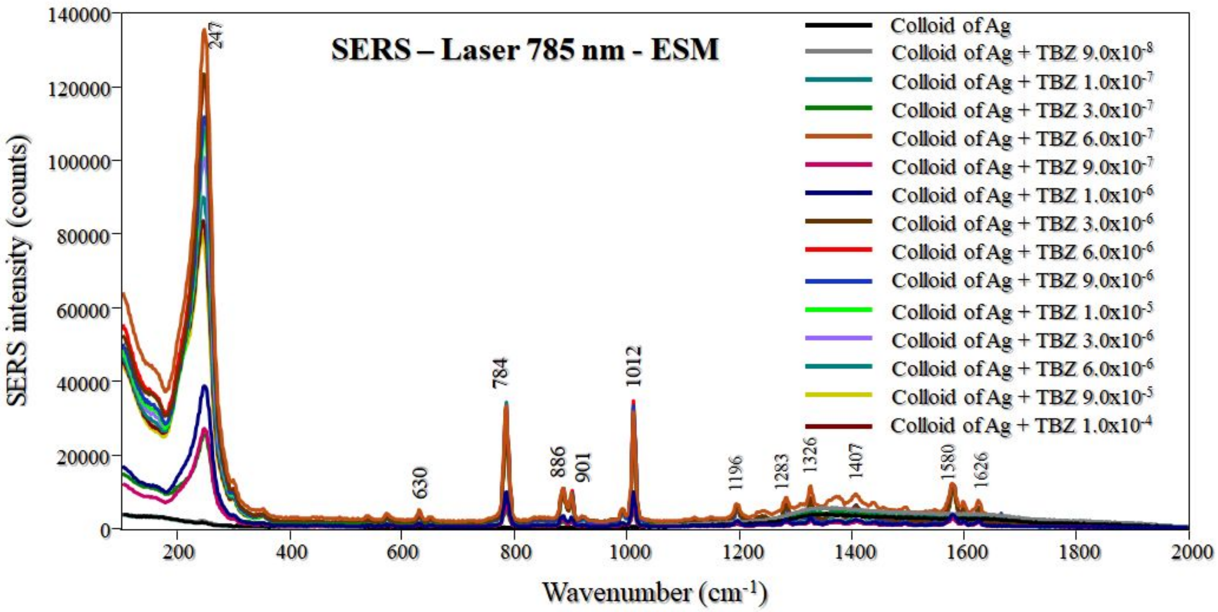

Figure S4: SERS spectra of TBZ (Ag colloid) from 9.0×10^{-8} to 1.0×10^{-4} mol/L. The spectra up to 6.0×10^{-7} mol/L were obtained with 10% laser power, and with 1% laser power from 9.0×10^{-7} mol/L and higher concentrations due to detector saturation. The spectra were plotted with baseline and offset corrections (from 100 to 2000 cm^{-1}). Laser line at 785 nm. **ESM method**.

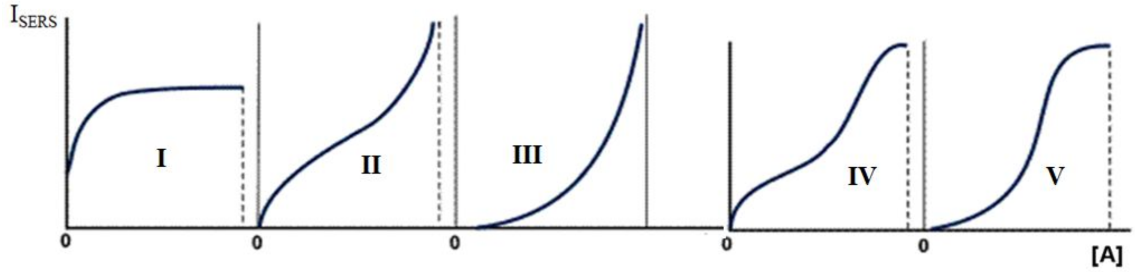

Figure S5: adsorption isotherms proposed by the B.D.D.T. model, adapted with permission from Brunauer *et al.*[1].

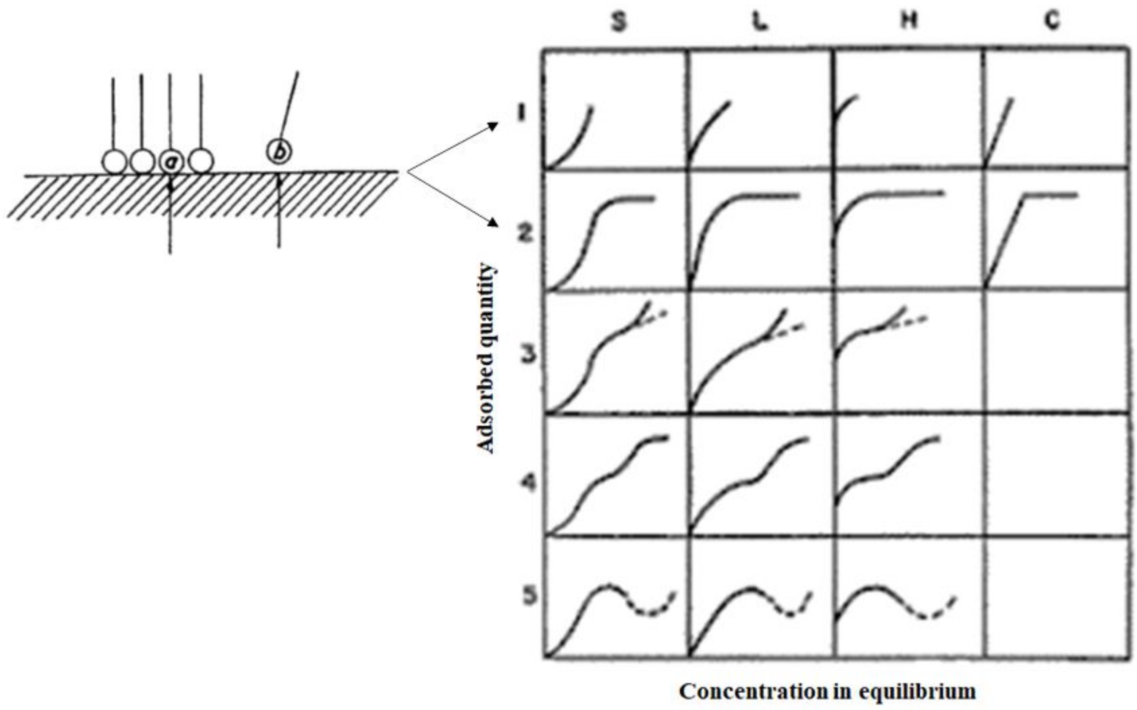

Figure S6: Giles *et al.* isothermal classification system.[2] Detail: schematic illustration of the conditions that favor the generation of isotherms S and L. Monofunctional polar solute on the polar substrate in a polar solvent. A solute molecule is more stable and adsorbed on "a", adjacent to other molecules already adsorbed, than on "b" itself. Result: isotherm S. Adapted with permission from Giles *et al.*[2].

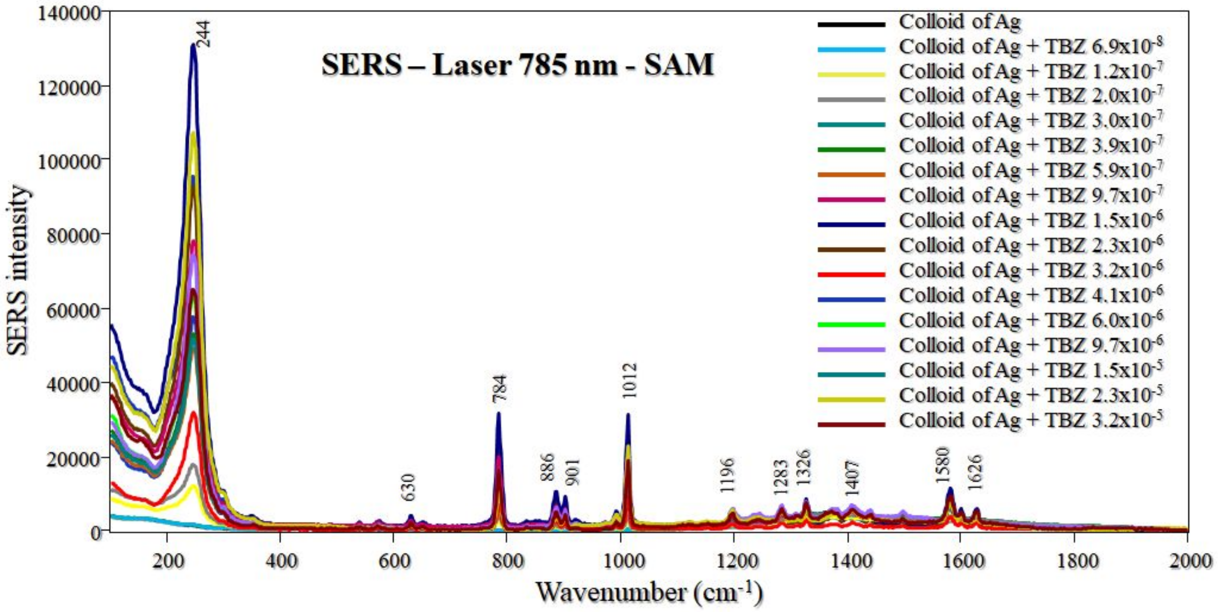

Figure S7: TBZ SERS spectra (Ag colloid) from 9.9×10^{-9} to 3.2×10^{-5} mol/L. The spectra up to 5.9×10^{-7} mol/L were obtained with 10% laser power, and with 1% laser power from 9.7×10^{-7} mol/L and higher concentrations due to detector saturation. The spectra were plotted with baseline and offset corrections (from 100 to 2000 cm^{-1}). Laser line at 785 nm. **SAM method**.

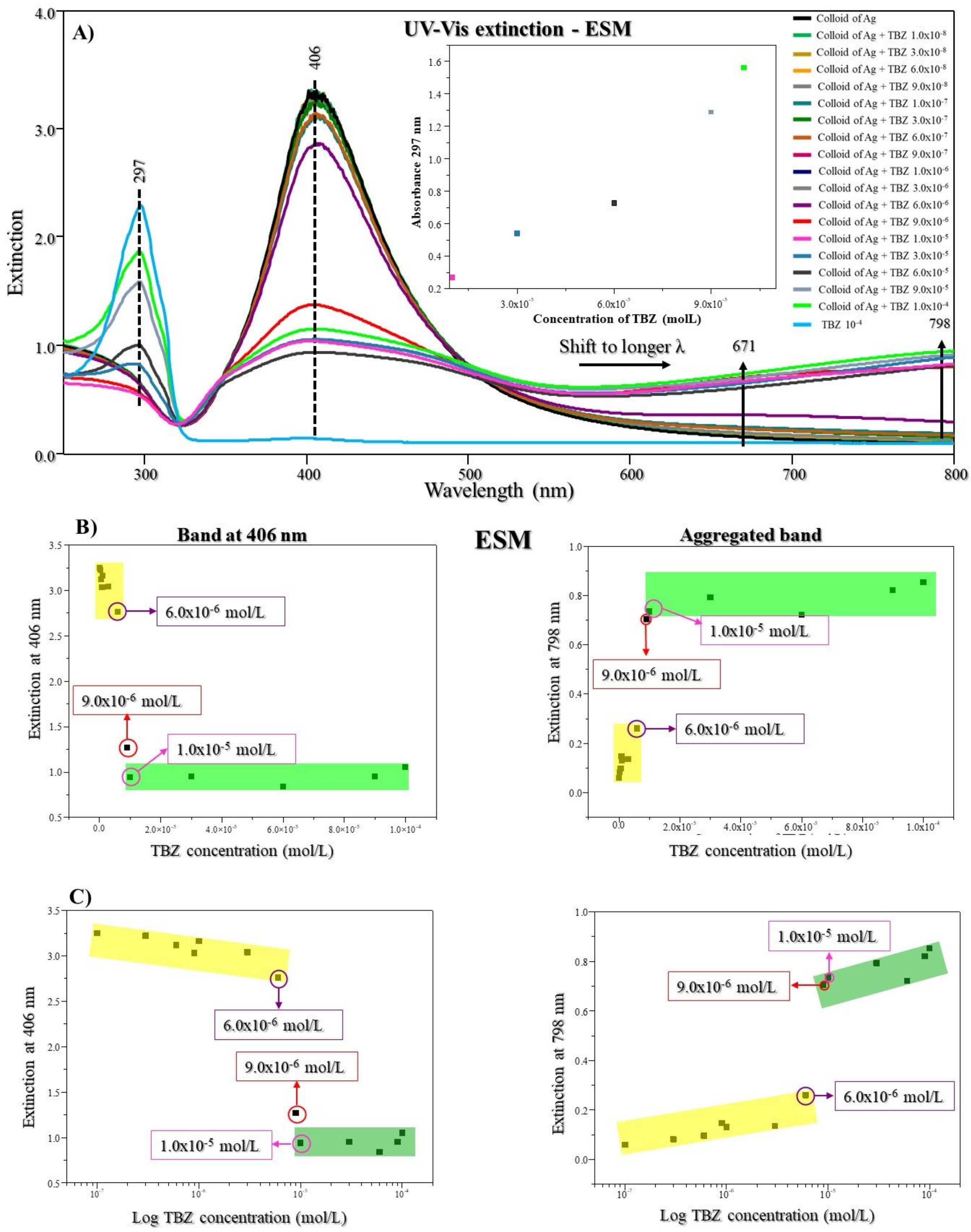

Figure S8: A) UV-Vis extinction spectra of Ag colloid in the absence and presence of TBZ from $1.0x10^{-8}$ to $1.0x10^{-4}$ mol/L. The baseline for the sample with Ag colloid was a quartz cuvette filled with ultrapure water. **B)** Extinction vs. TBZ concentration (mol/L) for the bands at 406 nm (isolated AgNPs or transversal plasma resonances of the aggregated AgNPs) and 798 nm (aggregated AgNPs). **C)** Extinction vs. Log TBZ concentration (mol/L) for the bands at 406 nm (isolated AgNPs or transversal plasma resonances of the aggregated AgNPs) and 798 nm (aggregated AgNPs). **ESM method**.

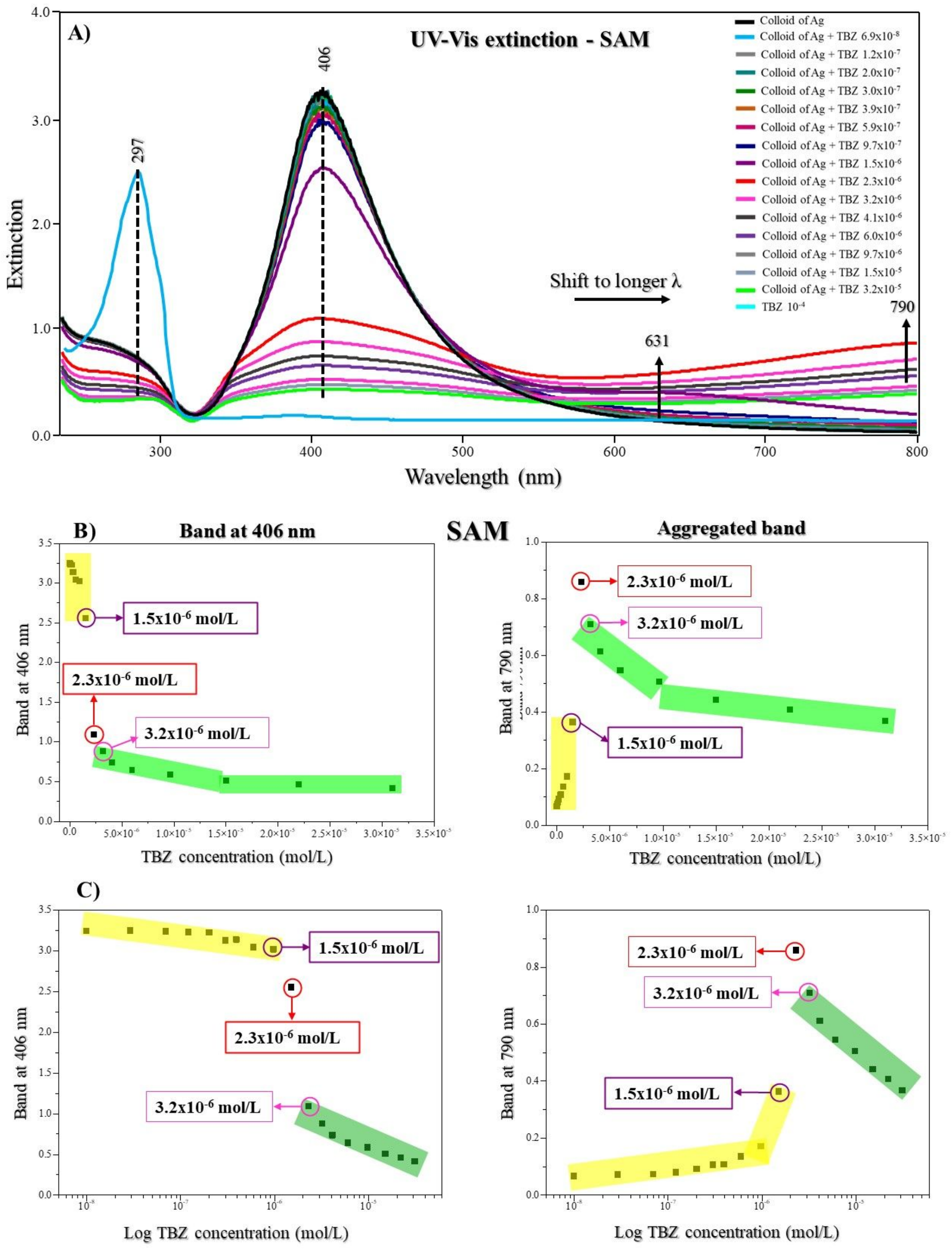

Figure S9: A) UV-Vis extinction spectra of the Ag colloid in the absence and presence of TBZ from $6.9x10^{-8}$ to $3.2x10^{-5}$ mol/L. The baseline for the sample with colloid of Ag: quartz bucket with ultrapure water. **B)** Extinction vs. TBZ concentration (mol/L) for the bands at 406 nm (isolated AgNPs or transversal plasma resonances of the aggregated AgNPs) and 790 nm (aggregated AgNPs). **C)** Extinction vs. Log TBZ concentration (mol/L) for the bands at 406 nm (isolated AgNPs or transversal plasma resonances of the aggregated AgNPs) and 790 nm (aggregated AgNPs). **SAM method**.

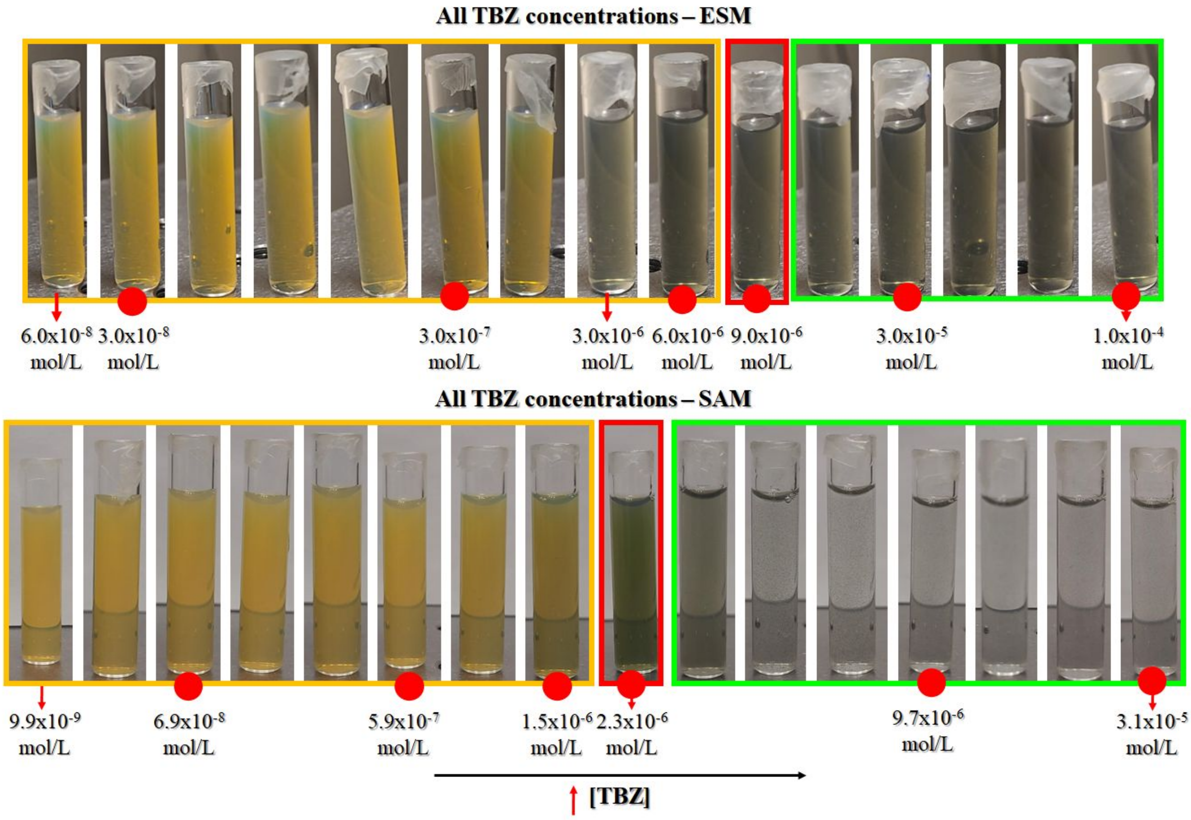

Figure S10: optical microscopy images of the Ag colloid for various TBZ concentrations in both methods (**ESM** and **SAM**). The circles in red refer to the TBZ concentrations used in the UV-Vis extinction spectroscopy and TEM microscopy.

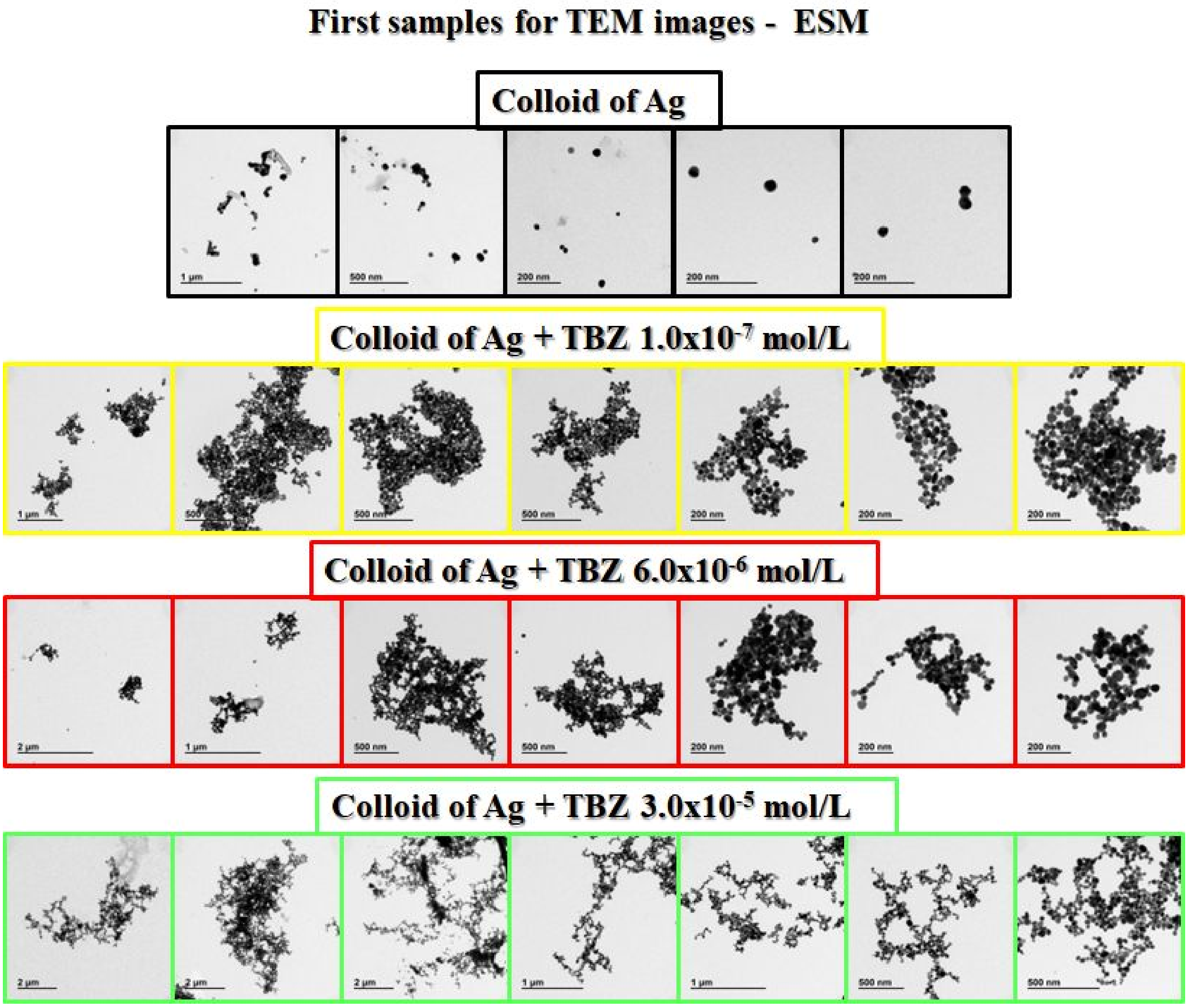

Figure S11: TEM images of Ag colloid in the absence and presence of TBZ at 1.0×10^{-7}, 6.0×10^{-6}, and 3.0×10^{-5} mol/L. **ESM method**.

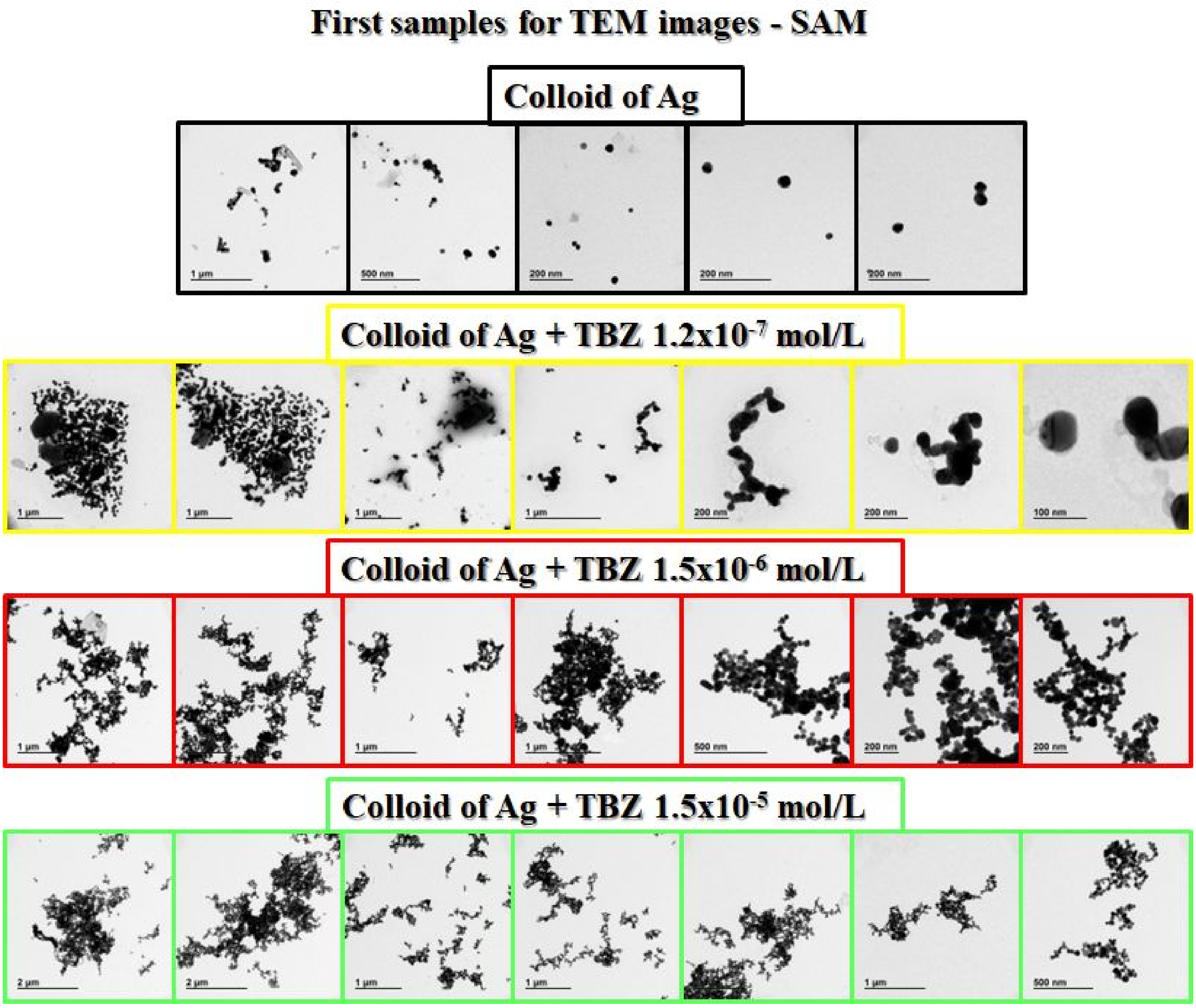

Figure S12: TEM images of Ag colloid in the absence and presence of TBZ at $1.2x10^{-7}$, $1.5x10^{-6}$, and $1.5x10^{-5}$ mol/L. **SAM method**.

References

(1) Brunauer, S.; Deming, L. S.; Deming, W. E.; Teller, E. On a Theory of the van Der Waals Adsorption of Gases. *J. Am. Chem. Soc.* **1940**, *62* (7), 1723–1732. https://doi.org/10.1021/ja01864a025.

(2) Giles, C. H.; MacEwan, T. H.; Nakhwa, S. N.; Smith, D. 786. Studies in Adsorption. Part XI. A System of Classification of Solution Adsorption Isotherms, and Its Use in Diagnosis of Adsorption Mechanisms and in Measurement of Specific Surface Areas of Solids. *J. Chem. Soc.* **1960**, *846*, 3973. https://doi.org/10.1039/jr9600003973.
